# Nursing Students’ Beliefs and Willingness to Implement Evidence-Based Nursing Practice at Umm Al-Qura University: A Descriptive Cross-Sectional Study

**DOI:** 10.7759/cureus.58521

**Published:** 2024-04-18

**Authors:** Manal H Abo Elmagd, Muna Alharbi

**Affiliations:** 1 Psychiatric and Mental Health Nursing, Umm Al-Qura University, Mecca, SAU; 2 Nursing, Umm Al-Qura University, Mecca, SAU

**Keywords:** nursing science, clinical nursing, saudi nursing student, evidence-based practice beliefs, evidence-based practice course

## Abstract

Background

It is essential to provide evidence-based practice (EBP) courses for undergraduate nursing students. For this reason, students’ beliefs and intentions to implement EBP should be measured to ensure that EBP courses are effective.

Aim

The aim of this study is to evaluate Saudi nursing students’ EBP beliefs (EBPB) and implementation before they enroll in an EBP course.

Methods

A descriptive cross-sectional study was conducted. Two scales were used: the EBPB scale and the EBP implementation (EBPI) scale. The questionnaire was available for completion a single time before the second-year nursing students were introduced to the EBP course.

Results

The study revealed that the mean age for students is 20 ± 0.83. Nearly two-thirds (61.54%) of students are female. It can be noted that 71.5% have not attended any EBP programs. Additionally, 65.38% of students understand the concept of EBP, and 68.46% are willing to apply EBP nursing care. Therefore, there is a positive correlation between the EBPB scale variable and the EBPI scale.

Conclusion

This study highlights the positive attitude of undergraduate nursing students toward EBP. Nursing education programs must place more emphasis on integrating EBP curricula into their teaching strategies, with a focus on developing students’ knowledge, skills, and values in EBP.

## Introduction

Evidence-based practice (EBP) is now the gold standard of healthcare in the nursing profession. It must be prioritized by healthcare organizations and universities. Thus, to meet the expectations of the healthcare system in the 21st century, healthcare professionals are expected to be competent in the application of EBP. Numerous academic initiatives to introduce EBP to undergraduate nursing baccalaureate education have been implemented to equip future nurses to meet this requirement. One goal of baccalaureate nursing education is to provide nurses with opportunities to develop the competencies necessary for EBP [1].

Integrating EBP into the daily practices of healthcare professionals can enhance the practice environment and patient outcomes. It is critical for nurses to expand their knowledge base, standardize their practice, and improve patient outcomes. To advance nursing knowledge and improve future nursing practice and patient outcomes, nursing students must be taught the importance of evidence-based information and how to access, appraise, and use this information correctly as needed [2].

EBP incorporates clinical experience, the most recent and best available research evidence, and patients’ specific values and circumstances [3]. This type of practice is vital for nurses and the nursing profession, as it delivers numerous benefits: it assists nurses in developing their body of knowledge, bridging the gap between nursing education, research, and practice, and regulating nursing practices [4]; and it improves clinical patient outcomes, enhances healthcare quality, and reduces healthcare costs [5]. It enables nurses to base their clinical decision-making on the most accurate and recent research findings.

Evidence-based implementation is defined as the utilization of EBP in clinical practice [6]. Previous research revealed that there is inadequate EBP implementation (EBPI) in nurses’ daily clinical practices [7-9]. Studies have shown that strengthening nurses’ beliefs about EBP can help them implement it. These beliefs include the nurse’s view of the worth and benefits of EBP and their perceived self-confidence in their own EBP knowledge and skills [6]. Nurses with a strong belief in EBP apply it more often than those with a weak belief [8].

In a previous study, both nursing educators and undergraduate nursing students reported strong beliefs in EBP but low levels of EBPI [10]. Along with faculty members, they provided opportunities for the development of EBP. Based on these findings, it is proposed that funding should be provided for the development and testing of interventions aimed specifically at encouraging EBPI in nursing educational settings [10,11]. Thus, the aims of this study are to evaluate Saudi nursing students’ EBP beliefs (EBPB) and implementation before they enroll in the EBP course and to use the data as a basis for further developing the course.

Research purpose

The purpose of this study is to evaluate Saudi nursing students’ beliefs and willingness to implement EBP at Umm Al-Qura University in Saudi Arabia.

Research question

What are Saudi nursing students’ beliefs and willingness to implement EBP?

## Materials and methods

Research design

A descriptive cross-sectional study was conducted.

Research setting

The current study was conducted in the faculty of nursing at Umm Al-Qura University, Saudi Arabia.

Research subjects

A convenient nonprobability sample of 130 second-year students - 80 female and 50 male students - participated in this study. This number represents 53.5% of the total number of students in the second year, which is 243 (163 female and 80 male students).

Ethical considerations

To ensure that the study adhered to ethical standards, ethical approval from the formal ethical committee at Umm Al-Qura University was obtained before the start of the study (approval number HAPO-02-K-012-2023-02-1444). The first page of the questionnaire explained the purpose of the study and described what contributing to the study would entail for the participants.

Confidentiality and the ability to withdraw at any time without any penalty were also explained. When students agreed to fill out and submit the anonymous questionnaire, this constituted their consent to participate in the study. Other than the anticipated inconvenience of the time spent completing the questionnaire, no actual risks were associated with this research. Data protection and security were ensured using password-controlled computerized databases.

Data collection

Two scales were used: the EBPB scale and the EBPI scale [12]. These scales have been used and tested for reliability and validity among nurses [13]. In addition, demographic data were collected, including age and sex. Three questions were added: Do you attend any programs related to EBP? Do you know the meaning of EBP? Are you willing to apply EBP in your nursing care practice?

The EBPB scale consists of 16 statements that measure individual beliefs about the value of EBP and the ability to implement it [12,13]. Respondents were asked to score the level to which they agree or disagree with the 16 statements by answering on a five-point Likert scale ranging from “strongly disagree” (1) to “strongly agree” (5). Examples of the statements are “I believe the care that I deliver is evidence based” and “I believe that EBP results in the best clinical care for patients.” The total score for the 16 questions ranges from 16 to 80 points. Two items (numbers 11 and 13) are reverse scored, and the scores for all items are added to give a total score. Higher scores and means reflect more positive beliefs about EBP.

To study nurses’ beliefs about EBP, we used four subscales of the EBPB scale, as defined by Estrada [14]: (1) knowledge beliefs, (2) value beliefs, (3) resource beliefs, and (4) time and difficulty beliefs. The items related to knowledge beliefs consist of knowledge of the steps of EBP, the measurement of outcomes, and the implementation to make changes in practices (n = 5). The value items include the belief that EBP results in the best clinical care and improves patient care (n = 5). Included in the resource items are access to the best resources and the ability to overcome barriers (n = 4). Time and difficulty belief items include the time EBP takes and whether nurses find EBP difficult (n = 2).

The EBPI scale consists of 18 statements to which participants respond on a five-point frequency scale [12,13]. The questions are linked to the actual use of EBP in professional practice and measure the essential components and steps of EBP. For example, “How often have you ‘critically appraised evidence from a research study?’ or ‘used evidence to change my clinical practice?’” The answers range from “not at all” (1) to “a great extent” (5). The scoring involves summing the scores for the responses to the 18 items to obtain a total score, which may range from 18 to 90. Higher total scores and means reflect a more frequent display of EBP behaviors and skills [13].

In accordance with the student plan at the selected university, students must study the course for EBP in the second year of their university academic studies. The questionnaire was available for completion at a single time before the second-year students were introduced to the course. In this study, the questionnaire was distributed through Qualtrics and sent to all students through email and social media for a higher recruitment rate and greater accessibility.

Data analysis

We used IBM SPSS Statistics for Windows, Version 24.0 (Released 2016; IBM Corp., Armonk, NY, USA) to analyze the study data. The statistical tests used include descriptive statistics such as frequencies, means, and SDs, as well as inferential statistics such as a t-test for independent samples and one-way ANOVA to investigate the correlations between the study variables.

## Results

Table 1 reveals that the students’ mean age was 20 ± 0.83. Nearly two-thirds (61.54%) of the students were female. It can be noted from this table that 71.54% had not attended an EBP program. Additionally, 65.38% knew the meaning of EBP, and 68.46% were willing to apply EBP nursing care.

**Table 1:** Percentage distribution of demographic characteristics of nursing students (n = 130) EBP, evidence-based practice

Variables	Number	Percentage
Age		
19 ≤ 20 years	25	19.23%
21 ≤ 22 years	105	80.77%
Mean ± SD	20 ± 0.83	
Gender		
Male	50	38.46%
Female	80	61.54%
Attendance at any program related to EBP		
Yes	37	28.46%
No	93	71.54%
Do you know the meaning of EBP?		
Yes	85	65.38%
No	45	34.62%
Willingness to apply EBP nursing		
Yes	89	68.46%
No	41	31.54%

Table 2 illustrates that 24.62% and 36.15% of the students reported strongly agree or agree, respectively, that they are sure of their competence to implement EBP. Additionally, 49.23% either strongly agreed or agreed that they know how to sufficiently implement EBP to make practice changes. Moreover, 21.54% strongly agreed and 37.69% agreed that they feel confident about their abilities to implement EBP where they work. Regarding the responses to the value subscale, the majority of the students (29.23% strongly agree and 40.77% agree) believed that EBP results in the best clinical care for patients. In addition, 32.31% strongly agreed and 35.38% agreed that evidence-based guidelines can improve clinical care. As for the resource access subscale, they believed that they could search for the best evidence to answer clinical questions in a time-efficient way (21.54% and 36.15% strongly agree and agree, respectively). In addition, 18.46% and 36.15% of the students reported strongly agree or agree, respectively, that they are sure they can access the best resources to implement EBP. Meanwhile, a segment of the students strongly agreed or agreed (19.23% and 32.31%, respectively) that EBP takes too much time.

**Table 2:** Percentage distribution of the EBPB subscale among nursing students (n = 130) EBP, evidence-based practice; EBPB, evidence-based practice belief

EBPB subscale	Strongly agree		Agree		Neither agree nor disagree		Disagree		Strongly disagree	
	No.	%	No.	%	No.	%	No.	%	No.	%
I: Knowledge belief										
2. I am clear about the steps of EBP.	22	16.92	43	33.08	23	17.69	36	27.69	6	4.62
3. I am sure that I can implement EBP.	32	24.62	47	36.15	33	25.38	15	11.54	3	2.31
10. I understand how to measure the outcomes of clinical care.	26	20	44	33.85	36	27.69	20	15.38	4	3.08
14. I know how to implement EBP sufficiently to make practice changes.	25	19.23	39	30	42	32.31	16	12.31	8	6.15
15. I am confident about my ability to implement EBP where I work.	28	21.54	49	37.69	36	27.69	14	10.77	3	2.31
II: Values belief										
1. I believe that EBP results in the best clinical care for patients.	38	29.23	53	40.77	30	23.08	8	6.15	1	0.77
4. I believe that critically appraising evidence is an important step in the EBP process.	30	23.08	55	42.31	32	24.62	10	7.69	3	2.31
5. I am sure that evidence-based guidelines can improve clinical care.	42	32.31	46	35.38	27	20.77	12	9.23	3	2.31
9. I am sure that implementing EBP will improve the care that I deliver to my patients.	31	23.85	59	45.38	29	22.31	9	6.92	2	1.54
16. I believe the care that I deliver is evidence based.	21	16.15	54	41.54	45	34.62	9	6.92	1	0.77
III: Resource access										
6. I believe that I can search for the best evidence to answer clinical questions in a time-efficient way.	28	21.54	47	36.15	38	29.23	14	10.77	3	2.31
7. I believe that I can overcome barriers to implement EBP.	20	15.38	47	36.15	48	36.92	12	9.23	3	2.31
8. I am sure that I can implement EBP in a time-efficient way.	20	15.38	46	35.38	48	36.92	14	10.77	2	1.54
12. I am sure that I can access the best resources to implement EBP.	24	18.46	47	36.15	40	30.77	15	11.54	4	3.08
IV: Time difficulties										
11. I believe that EBP takes too much time.	25	19.23	42	32.31	49	37.69	12	9.23	2	1.54
13. I believe EBP is difficult.	21	16.15	36	27.69	51	39.23	20	15.38	2	1.54

Table 3 reveals that 19.23% and 18.46% of the students did not at all generate a population, intervention, control, and outcomes (PICO) question about their clinical practice and critically appraised evidence from a research study. In addition, 21.54% and 23.85% of the students did not access the Cochrane Database of Systematic Reviews or the National Guidelines Clearinghouse, respectively. Furthermore, a number of the students did not at all or to a small extent (19.23% and 20.77%, respectively) read and critically appraise a clinical research study. Moreover, a segment of the students reported they do not at all or to a small extent (16.92% and 25.38%, respectively) informally discuss evidence from a research study with their colleagues.

**Table 3:** Percentage distribution of the EBPI scale among nursing students (n = 130) EBP, evidence-based practice; EBPI, evidence-based practice implementation; PICO, population, intervention, control, and outcomes

EBPI scale statement	Not at all		To a small extent		To some extent		To a moderate extent		To a great extent	
	No.	%	No.	%	No.	%	No.	%	No.	%
Used evidence to change my clinical practice	24	18.46%	19	14.62%	40	30.77%	20	15.38%	27	20.77%
Critically appraised evidence from a research study	24	18.46%	31	23.85%	37	28.46%	21	16.15%	17	13.08%
Generated a PICO question about my clinical practice	25	19.23%	36	27.69%	29	22.31%	24	18.46%	16	12.31%
Informally discussed evidence from a research study with a colleague	22	16.92%	33	25.38%	33	25.38%	31	23.85%	11	8.46%
Collected data on a patient problem	22	16.92%	27	20.77%	34	26.15%	25	19.23%	22	16.92%
Shared evidence from a study in the form of a report with more than two colleagues	28	21.54%	33	25.38%	33	25.38%	18	13.85%	18	13.85%
Evaluated the outcomes of a practice change	23	17.69%	28	21.54%	27	20.77%	35	26.92%	17	13.08%
Shared an EBP guideline with a colleague	31	23.85%	28	21.54%	35	26.92%	20	15.38%	16	12.31%
Shared evidence from a research study with a patient or family member	28	21.54%	26	20.00%	36	27.69%	24	18.46%	16	12.31%
Shared evidence from a research study with a multidisciplinary team	30	23.08%	27	20.77%	36	27.69%	26	20.00%	11	8.46%
Read and critically appraise a clinical research study	25	19.23%	27	20.77%	31	23.85%	34	26.15%	13	10.00%
Accessed the Cochrane Database of Systematic Reviews	28	21.54%	34	26.15%	38	29.23%	17	13.08%	13	10.00%
Accessed the National Guidelines Clearinghouse	31	23.85%	30	23.08%	40	30.77%	21	16.15%	8	6.15%
Used an EBP guideline or systematic review to change clinical practice	27	20.77%	29	22.31%	26	20.00%	25	19.23%	23	17.69%
Evaluated a care initiative by collecting patient outcome data	27	20.77%	27	20.77%	29	22.31%	29	22.31%	18	13.85%
Shared the outcome data collected with colleagues	27	20.77%	28	21.54%	27	20.77%	23	17.69%	25	19.23%
Changed practice based on patient outcome data	26	20.00%	34	26.15%	26	20.00%	23	17.69%	21	16.15%
Promoted the use of EBP to my colleagues	33	25.38%	23	17.69%	31	23.85%	27	20.77%	16	12.31%

In Table 4, the independent t-test was used to investigate the mean differences between male and female students in relation to the EBPB scale, its four subscales, and the EBPI scale. It reveals a significant difference between the male and female students’ means of the total EBPB scale and the “knowledge” and “values” subscales. Meanwhile, there are insignificant differences in the means of male and female students in relation to the total EBPI scale (p-value 0.56).

**Table 4:** Differences between male and female nursing students regarding EBPB and EBPI scales (n = 130) * indicates a significant p-value ≤ 0.05. EBP, evidence-based practice; EBPB, evidence-based practice belief; EBPI, evidence-based practice implementation

Variables	Male		Female		Mean difference	t-test	p-value
	Mean	SD	Mean	SD			
Knowledge domain	16.42	± 4.64	18.35	± 4.299	1.93	2.41	0.01*
Values domain	18.04	± 4.04	19.63	± 3.822	1.59	2.26	0.02*
Resources domain	13.96	± 3.63	14.42	± 3.381	0.46	0.74	0.46
Time difficulty domain	5.32	± 1.78	4.8	± 1.701	0.52	1.66	0.09
EBPB scale	53.74	± 10.43	57.21	± 9.460	3.47	1.95	0.05*
EBPI scale	28.98	± 19.44	35.1	± 17.870	6.12	1.83	0.56

## Discussion

Baccalaureate nursing students are the future of the nursing profession and play a critical role in promoting EBP. Undergraduate nursing students’ beliefs in and implementation of EBP have been the focus of recent studies. In recent years, EBP has become an integral aspect of healthcare. It is an approach in which clinical decision-making is based on the most current and valid evidence available. However, its implementation can be challenging, particularly for novice healthcare practitioners, such as nursing students.

This study aimed to examine the beliefs and implementation of EBP among nursing students before they attend the EBP course. Its goal was similar to that of previous studies, whose results provided an understanding of students’ attitudes, knowledge, and skills in EBP and indicated a starting point to develop effective strategies for EBP curricula [1]. This study confirmed previous studies’ findings that nursing students have a positive attitude toward EBP and recognize its importance in providing quality patient care. However, the implementation of EBP has been found to be challenging for nursing students [2].

In this study, nursing students were found to believe that EBP would enhance their critical thinking, increase their confidence in clinical decision-making, and improve patient care. They acknowledge the importance of using evidence-based resources, such as clinical practice guidelines, to guide their practice. This is similar to the results of another study, which found that nursing students have a good understanding of the process of EBP and the steps involved in evidence-based decision-making but lack the necessary skills to perform literature searches and critically appraise research evidence [15].

EBPI in nursing is crucial for providing safe, effective, and efficient care to patients. Undergraduate nursing students’ beliefs about EBP play an essential role in determining their acceptance and implementation of EBP in clinical settings. The findings of this study suggest that nursing students have positive beliefs toward EBP but face several barriers to implementing EBP in clinical practices. These findings are consistent with those of previous research that found that despite students’ positive attitudes toward EBP, many challenges exist in its implementation [10-16].

One important finding of this study is that nursing students are motivated to use EBP to improve patient outcomes. However, barriers related to time constraints and a lack of authority remain significant challenges. These findings are supported by previous studies that reported barriers such as a lack of time, knowledge, resources, and support from colleagues and managers [17-19]. Nursing students have reported that they often lack the necessary skills and knowledge to implement EBP, suggesting that nursing education programs should place more emphasis on the development and integration of EBP curricula in undergraduate programs.

The findings of this study additionally suggest that nursing students have positive beliefs about the relevance, usefulness, and applicability of EBP in clinical settings. This is consistent with previous studies that reported that nursing students have positive attitudes toward the potential of EBP to improve patient care [10-17]. These findings reinforce the importance of incorporating EBP education into nursing curricula to improve students’ knowledge about and attitudes toward EBP.

The results of this study suggest that nursing education programs can promote EBPI by creating a supportive environment that encourages its adoption. This involves providing students with the resources and support necessary to develop the skills and knowledge required to implement EBP. In addition, nursing education programs can foster students’ positive attitudes toward EBP by emphasizing its importance in improving patient outcomes and promoting the benefits of EBPI in clinical settings.

The implementation of EBP in nursing is crucial to ensuring the provision of high-quality patient care. The findings of this study suggest that undergraduate nursing students must be equipped with the necessary knowledge, skills, and attitudes to implement EBP in their clinical practice. The results of this study are consistent with previous studies that highlighted the need for nursing education to incorporate EBP as a core component of the undergraduate curriculum [20].

Previous studies reported that undergraduate nursing students often lacked the necessary skills and knowledge to implement EBP [21,22]. This study reinforces these findings, highlighting the need for nursing education programs to improve the way EBP is taught. The study results indicate that, generally, nursing students have positive attitudes toward EBP and recognize the importance of implementing EBP in their clinical practice. However, the participating students reported a lack of confidence in implementing EBP, which suggests that more support is required to build the necessary skills, knowledge, and confidence.

This study suggests that nursing education programs must provide more opportunities for students to learn and apply the EBP process in clinical settings. Providing exposure to clinical settings where EBP is routinely implemented, such as through clinical placements or internships, could enable nursing students to develop their skills in and confidence with EBP. Moreover, nursing educators must emphasize the importance of EBP by integrating it appropriately into the nursing curriculum, with a focus on developing and maintaining the necessary skills among students. The development of an EBP curriculum requires experienced staff, adequate resources, and commitment from institutional leadership.

Limitation

This study adopted a convenient sampling method that limited the generalization of the results.

## Conclusions

This study highlights the positive beliefs of undergraduate nursing students toward EBP. Nursing education programs must place more emphasis on integrating EBP curricula into their teaching strategies, with a focus on developing students’ knowledge, skills, and values in EBP. In addition, creating supportive environments that encourage EBPI in clinical settings is essential, including addressing time and resource constraints and facilitating collaboration among healthcare teams. The research respondents indicated a lack of confidence in implementing EBP, which suggests a need for further support and training. Overall, it is essential for nursing education programs to prioritize the development of a comprehensive EBP curriculum to ensure that nursing graduates are equipped to provide quality patient care based on the best available evidence.
